# In the hands of the beholder: Wearing a COVID-19 mask is associated with its attractiveness

**DOI:** 10.1177/17470218211037128

**Published:** 2021-08-11

**Authors:** Veronica Dudarev, Maria GM Manaligod, James T Enns, Rebecca M Todd

**Affiliations:** 1 Department of Psychology, The University of British Columbia, Vancouver, British Columbia, Canada; 2 Djavad Mowafaghian Centre for Brain Health, The University of British Columbia, Vancouver, British Columbia, Canada

**Keywords:** Microvalence, emotional appraisal, affective appreciation, affective devaluation

## Abstract

Protective facial masks reduce the spread of COVID-19 infection and save lives. Yet a substantial number of people have been resistant to wearing them. Considerable effort has been invested in convincing people to put on a mask, if not for their own sake than for those more vulnerable. Social and cognitive psychologists know that use and liking go both ways: people use what they like, and they like what they use. Here we asked whether positive attitudes towards facial masks were higher in those who had been wearing them longer. We asked participants in a diverse sample (*N* = 498 from five countries and more than 30 US states) to rate how attractive and emotionally arousing masks and other objects associated with COVID-19 were in comparison to neutral objects, as well as reporting on their mask-wearing habits. To confirm reliability of findings, the experiment was repeated in a subset of participants 8–10 weeks later. The findings show that regular use of protective masks was linked to their positive appraisal, with a higher frequency and a longer history of wearing a mask predicting increased mask attractiveness. These results extended to other COVID-related objects relative to controls. They also provide critical ecological validity for the idea that emotional appraisal of everyday objects is associated with our experience of using them. Practically, they imply that societal measures to encourage mask wearing may have contributed to positive emotional appraisals in those who put them on, whether due to personal choice or societal pressure.

## Introduction

Protective facial masks reduced the spread of COVID-19 infection and saved lives (Gandhi et al., 2020; Lyu & Wehby, 2020). Yet a substantial number of people have been resistant to wearing masks, especially younger people, who have been more likely to spread the disease because of their higher frequency of social contacts (Knotek et al., 2020). Moreover, some people have been resistant to wearing masks because of personal beliefs and ideological affiliations (Aratani, 2020; Beer, 2020; Karalis, 2020; Knotek et al., 2020; Miller & Brueck, 2020).

A potentially useful tool in the effort to increase behaviours such as mask wearing during a pandemic comes from research in the cognitive sciences, particularly on the bidirectional relations between our actions towards and our emotional appraisals of objects in our environments. There is a large literature documenting that visual attention is involuntarily drawn to images of objects that we find emotionally rewarding (Anderson, 2016; Chelazzi et al., 2013; Failing & Theeuwes, 2018). At the same time, there is a growing literature documenting that the arrow of influence runs in the other direction too: Merely attending to or acting on an object increases its emotional salience (Fenske & Raymond, 2006; Hayes et al., 2008; Peck & Shu, 2009; Schonberg et al., 2014; Streicher & Estes, 2015; Wispinski et al., 2020), while ignoring an object to select another one reduces it (De Vito & Fenske, 2018; Fenske et al., 2004; Griffiths & Mitchell, 2008; Kiss et al., 2007; Raymond et al., 2005) even in preverbal infants (Silver et al., 2020).

Based on such findings, the *usage-positive appraisal hypothesis* proposes that the emotional appraisal of objects faithfully mirrors the degree to which those objects are attended to and acted upon. Yet, very few studies have investigated this hypothesis in real-life circumstances. In fact, research on emotional appraisal of natural stimuli has traditionally focused on objects and scenes that possess strong affective valence, such as snakes and spiders on the negative side, and celebrations and flowers, or erotic imagery, on the positive side. Stimuli that lie between those extremes on average, or may vary in valence between individuals have been traditionally considered “neutral.” However, there is now evidence that mundane objects give raise to subtle, yet detectable and consistent valences, or *microvalences* (Lebrecht et al., 2011, 2012). Moreover, research has shown these subtle valences to influence people’s real-world choices (Lebrecht & Tarr, 2012). The theory of microvalence suggests that these affective properties associated with mundane items are accumulated with every encounter with the object, resulting in slight preferences or anti-preferences. As every person’s history of interacting with an object is more or less unique, and as microvalences are shaped by many other forms of associations as well (Manaligod, 2020; Roos et al., 2013; Lebrecht et al., 2012), investigation of how microvalences arise is especially challenging. We suggest that one way to examine the subtle rise and fall of affect in everyday objects is by examining the use of novel, unfamiliar objects.

Indeed, the familiarity of an object itself plays into its microvalence. The time course of the emotional appeal of an object is best described by an inverted U-shaped function (Biederman & Vessel, 2006; Isik & Vessel, 2019). Humans are sometimes referred to as “infovores,” because the acquisition of a new understanding is inherently pleasurable (Kang et al., 2014). The click of comprehension is thought to be associated with the release of endorphins to the association areas of the brain, as it makes rich connections with mnemonic information. But this search for the pleasure of encountering new knowledge only describes the short-lived, ascending, part of the inverted-U function. Following that, a decline in preference often sets in through a process referred to as habituation, such that highly rewarding stimuli no longer elicit the same degree of reward (Berlyne, 1970; Biederman & Vessel, 2006; Isik & Vessel, 2019).

But where do we find a real-life situation in which people encounter novel objects en masse? The circumstances of the COVID-19 pandemic, where early in the pandemic people were faced with the choice of using or not using protective masks, offered us precisely such an opportunity. The pandemic introduced objects into our daily lives for which the majority of people had no firsthand experience: protective face masks. This allowed a test of whether one’s practice of wearing protective masks—or not wearing them—was associated with one’s attitudes towards the masks, keeping previous experience under greater control than for most other objects.

An ecological and observational study such as this will necessarily lack many of the controls one expects to see in a laboratory study. We believe these limitations in being able to make strong causal statements at the conclusion of the study are offset by the benefits that come from studying human behaviour “in the wild.” In our view, causal inference and ecological validity necessarily trade off with one another in the design of the study, with the balance in our field typically favouring increases in the strength of causal logic, but at the expense of ecological validity. Here, by favouring ecological validity over rigorous control, we deliberately intend to address this imbalance in the way studies on the links between actions and emotion are typically conducted. After all, what benefits do strong causal statements provide without some understanding of whether the same variables are ever in similar relations in the natural world?

With this perspective in mind, we looked for some suitable natural objects that might serve as appropriate controls for the perception of the protective masks we intended to study. We settled on recreational masks used in diving and skiing, as their familiar use had likely not been affected by the pandemic. We hypothesised that the association between use and emotional appraisal would be more pronounced for the objects made novel by the pandemic (i.e., protective masks) than for other objects that were previously familiar (i.e., recreational masks). As another control, we compared protective masks with other objects that were now more salient, but which, we assumed, were quite familiar to most people prior to the pandemic: latex gloves and hand sanitizers. Following the same reasoning, we expected that the effect would be strongest for masks than for gloves and hygiene products.

**Figure 1.**
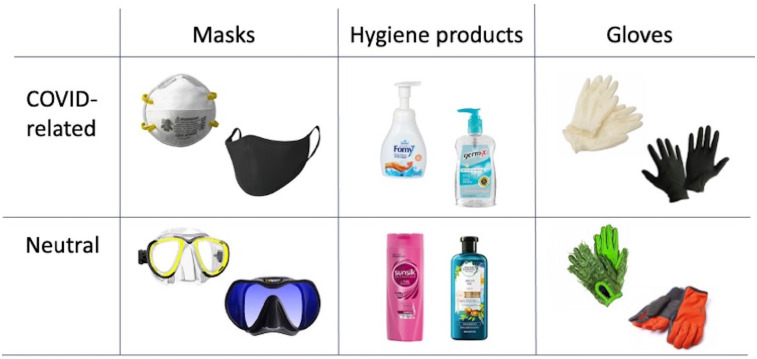
Examples of product images that were rated for attractiveness and emotional intensity in this study. The two exemplars shown in each category illustrate the variation in average attractiveness (low on the left, higher on the right), as indicated by the participants as a whole.

The study was conducted online in the July–August 2020 (first wave) and October 2020 (replication) using participants’ personal devices (i.e., computers, tablets) in a setting of their own choice, and over which we had no control. The experimental software we designed presented participants with product images to evaluate with subjective ratings, using the cover story that the study was intended to measure the aesthetics of various common consumer products. The products included the objects shown as examples in Figure 1, half of which were selected by us to be COVID-19 related and half of which were unrelated. Participants provided 7-point ratings of attractiveness and emotional intensity for each item, presented in a different random order for each participant. We also asked participants whether, how often, and for how long they have been wearing protective face masks for COVID-19, along with a battery of other questions in an exit survey. To examine test–retest reliability of our measures, we repeated the experiment in a subset of participants 2–2.5 months later.

Because we wanted to maximise the range of mask use and experience, we recruited a majority of the sample from 19 US states where, at the time of testing (July–August 2020), wearing a mask was still optional. In our sample (*n* = 493), 80.3% reported wearing protective masks “very often,” 13.2% reported wearing them “sometimes,” and the remaining 6.5% reported wearing masks rarely or never. 8.7% participants reported having worn protective masks previous to the pandemic.

## Method

### Participants

Our goal was to detect a statistical interaction in ratings made by participants in the following mixed-factor analysis of variance design: two levels of image (COVID-related, neutral) in a within-participant factor × 3 levels of mask use (never, occasional, frequent) in a between-participant factor. We estimated that a sample of 72 would be sufficient to reveal a small effect ( $\eta_{p}^{2}=0.06$ ) with power of .80, if there was an equal distribution of participants between groups. By July 2020, however, the assumption of equal distribution of participants in three groups was becoming increasingly unrealistic because of the rapid adoption of protective masking in many regions of North America. We therefore began by collecting data from 100 participants to first estimate a rough proportion of people who had not yet started wearing protective masks. Participants aged 18 years and above were recruited on prolific.co (www.prolific.co), without any other demographic or geographic restrictions. Out of 101 participants who completed the study, only 10 (9.9%) were not wearing masks or were wearing them rarely. We used these data to estimate that to get a minimum of 23 participants per group, we would need to recruit at least 400 more participants. We also limited this second round of recruitment to people from those US states where mask wearing was not mandatory at the time of the study (early August 2020).

Of the 498 participants across the two samples, 492 participants completed the product image rating task, 384 participants answered all survey questions without omissions (see more details below), and 379 completed both. The sample was well balanced in gender (45.6% male, 51.3% female participants) and ranged in age from 18 to 78 years (mean 33.7, median 31); 45 participants (9.2%) had been diagnosed with COVID-19 or suspected they had it. Exclusion of those participants did not change any of the results that are reported.

To test reliability of our measures and findings, we contacted all people who participated in the first wave of the study inviting them to take part in the second wave; 341 participants responded, 330 of them answered all the questions and completed the rating task.

### Materials

Images of masks, gloves, and personal hygiene products were used as stimuli, with 20 stimuli in each category. Half of the stimuli were COVID-related (medical or cloth face masks, latex gloves, hand sanitizers) and the other half were neutral (diving or skiing masks, cloth gloves, shampoo). The items were downloaded from consumer websites. Aiming to represent the diversity of products that people encounter, we selected both relatively ugly and attractive items for each category. All items were then pre-rated by the study authors and research assistants to determine their general levels of attractiveness (see Figure 1). We selected items that were rated as most and least attractive, to represent the full spectrum of attractiveness. Selected stimuli were presented to participants in random order.

The study was programmed in PsychoPy3 (Pierce et al., 2019) and Qualtrics and distributed via Pavlovia.org.

### Procedure

Participants were asked to rate stimuli on two 7-point scales: attractiveness and emotional intensity. For the attractiveness scale, the poles were labelled “ugly” and “attractive.” Emotional intensity rating was phrased as the strength of feeling towards an object, ranging from “weak” to “strong.” Participants were told to rate the strength of the feelings, either positive or negative, that they experienced when viewing each item. The order of questions was randomised across stimuli.

After the rating task, participants were asked a number of demographic information questions, as well as questions about practices and experiences with COVID and COVID-related equipment (masks, gloves, sanitizers). The key questions were (1) how often did you use protective face masks before 2020? (2) how often do you use masks now? and (3) when did you start using masks? In addition, a range of sociocultural, situational, and demographic measures were collected. The full list of questions is provided in the Supplementary Material. Out of 498 participants, the first 101 were presented with only a subset of these questions, 397 participants were presented with the full survey, and 384 of them answered all its questions.

The entire testing session took no longer than 20 min, and participants were paid 2 GBP. The study was approved by the Behavioural Research and Ethics Board at the University of British Columbia (approval number H20-02162).

## Results

### Masks are more attractive to those who use them more

Our main hypothesis was that subjective ratings of the attractiveness of protective masks would be predicted by their more frequent use. We tested this hypothesis in two ways. First, we compared attractiveness ratings for protective masks in participants who were wearing masks very often, sometimes, and rarely or never, while using recreational (diving and skiing) masks as control stimuli. Second, we tested whether the length of time an individual had regularly worn a mask predicted ratings of mask attractiveness.

**Figure 2.**
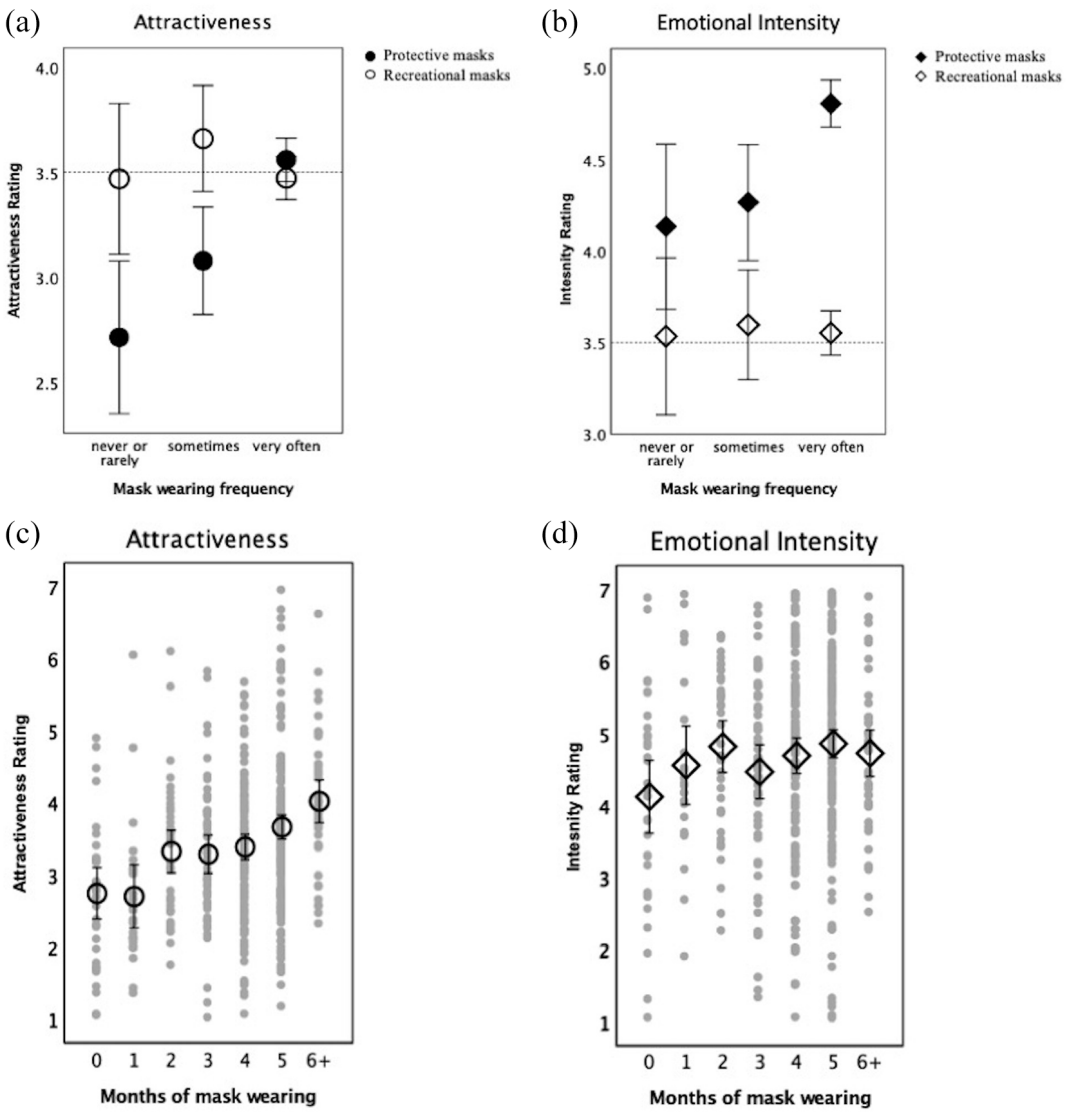
Mean rating of attractiveness (a, c) and intensity (b, d) on a 7-point scale for protective facial masks (in black) and recreational masks (in white) as a function of the frequency (a, b) or duration (c, d) of mask wearing. Error bars show 95% confidence intervals around each mean estimate. Dashed lines mark the middle of the 7-point scale. In panels (c) and (d), grey dots show participants’ individual data points.

Figure 2a shows that, in the eyes of frequent mask-wearers, protective masks were as attractive on average as recreational masks, *t*(395) = 1.7, *p* > .08, and much more attractive than in the eyes of those who wore the masks only sometimes or not at all, *ts* > 3.5, *ps* < .002. The latter, in contrast to frequent mask-wearers, rated protective masks as much less attractive than recreational ones, *ts* > 4, *ps* < .001. These planned comparisons were supported by a mixed model ANOVA on attractiveness ratings, with frequency of wearing masks (between participants) and mask type (protective vs. recreational, within-participant) as independent variables. There were main effects of both mask wearing frequency *F*(2, 490) = 3.65, *p* = .027, $\eta_{p}^{2}=.015$ , and mask type, *F*(1, 490) = 31.2, *p* < .001, $\eta_{p}^{2}=.06$ . These were qualified by a significant statistical interaction between mask wearing frequency and mask type, *F*(2, 490) = 20.6, *p* < .001, $\eta_{p}^{2}=.078$ .

The test of whether the duration of regular mask-wearing predicted participants’ ratings of mask attractiveness was conducted using linear regression, with the main predictor being the number of months an individual had regularly worn a mask and the attractiveness ratings for recreational masks serving as a covariate. Figure 2c shows that the least positive ratings came from participants who had never worn protective masks or started wearing the previous week (*M* = 2.7, *SD* = 1), followed by ratings by participants who started wearing the masks during the pandemic (*M* = 3.4, *SD* = 1). The highest ratings were given by people who had worn the masks before the pandemic (*M* = 4, *SD* = 0.96). Furthermore, ratings increased linearly with time, as supported by a significant linear trend over the seven levels of time, β = .17, *t*(491) = 7.04, *p* < .001, while controlling for ratings for recreational masks, β = .51, *t*(491) = 13, *p* < .001. The overall model fit was *R*^2^ = .33.

### Masks evoke more emotional intensity in those who use them more

After viewing each product image, participants not only rated its attractiveness, but they also rated how emotionally intense it was. Participants were told to rate the strength of the feelings, either positive or negative, that they experienced when viewing each item (see Methods section). We submitted intensity ratings to the same analyses as described for mask attractiveness.

Figure 2b shows that frequency of mask use was associated with intensity ratings for protective and recreational masks in a manner that was similar to attractiveness ratings. Overall, the more often people wore masks, the more strongly they felt about protective, but not recreational masks. There was a main effect of mask type, *F*(1, 490) = 71.97, *p* < .001, $\eta_{p}^{2}=.128$ , and a trend for a main effect of mask-wearing frequency, *F*(2, 490) = 2.7, *p* = .066, $\eta_{p}^{2}=.011$ . These were qualified by an interaction between mask wearing frequency and mask type, *F*(2, 490) = 8, *p* < .001, $\eta_{p}^{2}=.032$ . While all participants, regardless of their mask wearing practices, rated protective masks as evoking more intense feelings than recreational masks, *ts* > 2.2, *ps* < .032, those who wore the masks very often rated the protective masks as more emotionally intense then infrequent wearers, *ts* > 2.5, *ps* < .015. For recreational masks, there were no differences between participants, *ts* < .3, *ps* > .7.

Figure 2d shows that the longer people had regularly worn masks, the more strongly they felt about them. Participants who had never worn the masks or started just recently rated masks as less arousing (*M* = 4.1, *SD* = 1.4) than people who have been using masks for some time (*M* = 4.7, *SD* = 1.3), *F*(2, 490) = 3.4, *p* = .035, $\eta_{p}^{2}=.014$ . When we used duration in months as a predictor of intensity ratings for protective masks, while controlling for intensity ratings for recreational masks, there was a significant linear trend, β = .073, *t*(491) = 2.19, *p* = .029, *R*^2^ = .196.

We also tested for gender differences in this pattern of results. Although females reported wearing protective masks more frequently than males, $\chi^{2}$ (2) = 7.2, *p* = .028, attractiveness and emotional intensity ratings did not differ between the genders, *ps* > .06, and neither was the interaction between frequency of use and item type (COVID-related vs. not) modulated by it, *ps* > .09. As for duration of mask-wearing, again, gender had no significant effect on either attractiveness or emotional intensity ratings, *ps* > .07. For attractiveness, there was an interaction between duration, in months, and gender, β = .012, *t*(491) = 2.39, *p* = .017. Although both male and female participants demonstrated an increase in attractiveness ratings the longer they were using the masks, for females the slope was steeper than for male participants. For emotional intensity, adding gender rendered the effect of duration to be insignificant, β = .084, *t*(491) = 1.83, *p* = .068, with no main effect or interaction with gender, *ps* > .07.

### Interim discussion of protective vs. recreational masks

The data so far suggest that attractiveness and intensity ratings of protective masks are positively associated with the frequency and duration of their use. At the same time, parallel results for skiing and diving masks, which are not associated with COVID, showed no association between emotional appraisal and the frequency and duration of wearing protective masks. But what is it that makes protective masks special?

Our reasoning, following from the literature on object use and liking, was that the positive effects of usage on attractiveness would be most pronounced for objects that were newly introduced at the onset of the pandemic, namely, protective masks. We reasoned that previously-used objects, such as work gloves and hand sanitizers, might show a weaker or even no association with the pandemic-induced experience. This is why, in addition to asking participants about their mask-wearing practices, we asked analogous questions about the use of latex gloves and hand sanitizers to examine the effects of use on attractiveness and emotional intensity for each category specifically.

### Latex gloves and hand sanitizers also increase in attractiveness and intensity with use

Table 1 shows the frequency with which participants used latex gloves and hand sanitizers during and before the pandemic onset. The data show that in 2020 hand sanitizers were used with frequency comparable to the use of protective masks. However, notably, more than half of the participants had been using sanitizers before pandemic onset, meaning that hand sanitizers were not novel. In contrast, latex gloves had not been regularly used before the pandemic, and only a subset of the participants (one-third) were using them in 2020.

**Table 1.** Frequency and relative frequency of use reported for protective masks, latex gloves, and hand sanitizers.

	Protective masks, *N* (%)		Latex gloves, *N* (%)		Hand sanitizers, *N* (%)	
	Before 2020	Since pandemic onset	Before 2020	Since pandemic onset	Before 2020	Since pandemic onset
Rarely or never	449 (91.2%)	32 (6.5%)	420 (85.3%)	329 (66.9%)	189 (38.4%)	64 (13.1%)
Sometimes	22 (4.5%)	65 (13.2%)	54 (11%)	110 (22.4%)	234 (47.6%)	132 (26.8%)
Very often	21 (4.3%)	395 (80.3%)	18 (3.7%)	53 (10.8%)	69 (14%)	296 (60.2%)

**Figure 3.**
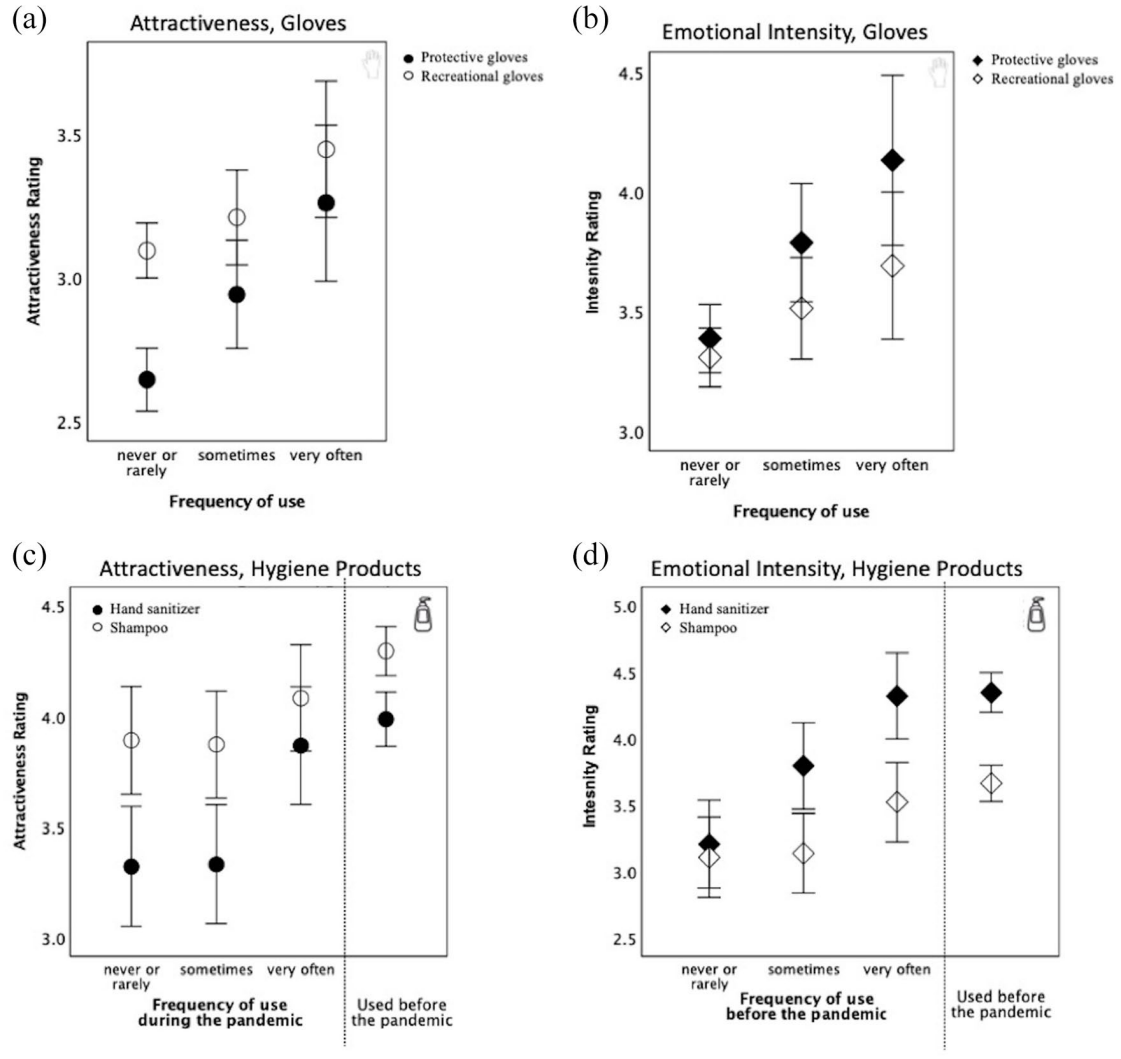
Mean rating of attractiveness (a, c) and intensity (b, d) on a 7-point scale for gloves (a, b) and personal hygiene products (c, d) as a function of the frequency of their use. COVID-related items (protective gloves and hand sanitizers) are shown in black colour, COVID-unrelated items (recreational gloves and shampoo) in white. Error bars show 95% confidence intervals around each mean estimate.

We tested the association between frequency of use and emotional ratings with four ANOVAs; one for each combination of image-type (gloves, personal hygiene) and rating-type (attractiveness, intensity). Each ANOVA examined the factors of frequency of use (rarely, sometimes, often) and COVID relevance (related, unrelated). Figure 3a and b shows that latex gloves were rated lower on attractiveness and higher on emotion intensity scales than COVID-unrelated gloves, main effect *F*(1, 490) = 33.2, *p* < .001, $\eta_{p}^{2}=.064$ and *F*(1, 490) = 17.6, *p* < .001, $\eta_{p}^{2}=.035$ , respectively. But, the more frequently participants used the gloves, the higher they rated them compared to recreational gloves on both attractiveness, interaction *F*(2, 490) = 8.86, *p* < .001, $\eta_{p}^{2}=.035$ , and emotional intensity, interaction *F*(2, 490) = 3.35, *p* = .036, $\eta_{p}^{2}=.013$ . Crucially, for those who were using the gloves very often, the difference between latex and recreational gloves was minimal for attractiveness, and latex gloves were more exciting than neutral ones, *F*(2, 490) = 3.1, *p* = .045, $\eta_{p}^{2}=.013$ for attractiveness, *F*(1, 490) = 3.35, *p* = .036, $\eta_{p}^{2}=.013$ for intensity.

A similar pattern emerged for personal hygiene products. There was a main effect of product type, with COVID-related sanitizers rated lower on attractiveness and higher on emotional intensity than COVID-unrelated shampoo, *F*(1, 490) = 74, *p* < .001, $\eta_{p}^{2}=.13$ for attractiveness and *F*(1, 490) = 17.6, *p* < .001, $\eta_{p}^{2}=.035$ for intensity. There was also a main effect of frequency of use, *F*(2, 490) = 15.7, *p* < .001, $\eta_{p}^{2}=.06$ and *F*(2, 490) = 7.7, *p* = .001, $\eta_{p}^{2}=.031$ , respectively. The interaction was significant for emotional intensity, *F*(2, 490) = 7.2, *p* = .001, $\eta_{p}^{2}=.029$ , but not for attractiveness, *p* > .13. It is important to bear in mind that according to the participants’ reported frequency of use of objects before the pandemic, hand sanitizers had been used regularly by about one half of the participants. This stands in contrast to protective masks and gloves, which had been used much less frequently. We reasoned that for the participants for whom hand sanitizers were familiar, the continued use during the pandemic would likely not have added to the already-established valence of these products. Thus, the correlation we hypothesised between product type (COVID-related versus neutral) and frequency of use should be stronger for novel than for familiar products, as described in the introduction. Therefore, we factored in previous use of hand sanitizer by adding another level to the between-participants factor of frequency of use. This analysis revealed an interaction for attractiveness as well, *F*(3, 489) = 3.02, *p* = .029, $\eta_{p}^{2}=.018$ . Figure 3c and d shows that all participants rated shampoo as more attractive and less arousing than hand sanitizers. Yet, following the same pattern as was revealed for gloves and masks, people who were using hand sanitizers more often tended to see them as equally attractive as shampoos, and considerably more exciting.

### Interim discussion of personal hygiene products and latex gloves

One of the two non-mask categories (personal hygiene products) had been used extensively by participants before the onset of the pandemic whereas the other one (latex gloves) had not. Latex gloves and protective masks were both products that were generally familiar to participants prior to the pandemic. However, the frequency of use data we collected showed that they had not been part of most participants’ everyday lives. Because of this infrequent use, we expected their attractiveness and intensity ratings to be similar to the pattern for protective gloves: the more frequently one reported using gloves, the higher their ratings. And this is indeed what the data showed. This finding for latex gloves therefore further bolsters our hypothesis regarding use and liking for novel objects. Both masks and gloves, which had not been used very often before the pandemic, showed positive associations between frequency of use and emotional appraisal. Other products, such as hand sanitizers, were reported to be used more often before the pandemic by some participants and not by others. Here, the results showed that participants who had not used hand sanitizers previously rated their emotional appraisal in a similar way to their ratings for protective masks. Thus, we have three instances so far in which the novel use of items under natural circumstances is associated with an increase in their positive emotional appraisal.

### What factors mediate the relationship between object use and its emotional appraisal?

Although attractiveness and intensity ratings both showed a strong association with object use, with more frequent and longer use predicting more positive and stronger feelings, these data alone do not indicate what is driving this relationship. It is possible that the more frequent use of an object contributes to its appeal, but it is also possible that the aesthetic appeal of the object contributes to its more frequent use. It is also possible that a third set of factors, such as a participant’s political leaning to the right or left, or the requirements of mask wearing in workplace conditions, contribute to this relationship. Since this is an observational study, and not a controlled experiment, it is impossible to attribute direct causality to any of these factors. Nonetheless, a full examination of the individual differences gleaned from our demographic and attitude data helped to uncover some of the potential factors at play in the relationship between an object’s use and its emotional appraisal.

#### Demographic and attitude survey data

After completing the image rating task, participants completed a survey that included a range of sociocultural, situational, demographic measures, as well as questions related to participants’ practices of protection against COVID (use of facial masks, sanitizers, latex gloves). The complete list of 54 questions is given in the Supplementary Material. The questions included participants’ age, gender, income, profession, cultural background, religion, and preferred news sources (Whitman et al., 2018). In addition, there were questions about participants’ political leanings and interest in politics. Participants were also asked to indicate whether they considered themselves or their close ones to be vulnerable and whether they had been diagnosed with COVID-19. Several questions focused on situational factors that might expose them to the virus, such as working away from or in their own homes and how frequently they still met people in person (Fluharty & Fancourt, 2020; Wright et al., 2020). As a first step in using this information, we subjected these data to a principal component analysis (PCA), with the goal of discovering latent constructs that might be useful in better understanding the relationship between mask use and their emotional appraisal.

Our choice of the analysis—PCA—was dictated by the aim of summarising the demographic and situational data in the most parsimonious way. The dataset offered good opportunity for that, Bartlett’s test of sphericity, $\chi^{2}$ (325) = 2,860, *p* < .001, KMO measure of sampling adequacy .704. To account for the possibility of large correlations between components to be extracted, we also tested Principal Axis Factoring with Direct Oblimin and Promax rotations (in turn). However, the correlations between the factors did were small (*rs* < .12), suggesting that the orthogonal solution is the most parsimonious.

A total of 384 participants completed the survey. Assuming an agnostic approach, we began by entering all the variables we possibly could to the PCA,^1^ with the following exceptions. Several questions were omitted from all participants because they were (a) measured on nominal scales (e.g., ethnicity, news sources), (b) their answers were conditional on other questions (e.g., religious practices if one identified as a believer), or (c) the questions were directly related to the use of masks and other protective equipment. This left 26 items for the analysis. To account for differences in measurement units between these items, correlation-based PCA was used.

We first examined the Scree plot showing the relationship between goodness of fit and 10 potential candidate dimensions (see Supplementary Figure 1). The inflection in the curve pointed to three primary components, which combined accounted for 34.6% of the total variance in the data. Table 2 highlights the seven items with the highest loadings for each of the three components. We interpreted these components as (1) suspicion/belief in the danger of COVID (13.5% of variance), (2) interest/disinterest in politics (12.4% of variance), and (3) low/high situational exposure to COVID (8.7% of variance). It is worth noting that the two items contributing most strongly to the first component was a conservative political orientation and scepticism or suspicion about the danger of COVID-19. It is also notable that having an interest in politics constituted a separate dimension, independent from the political orientation and suspicion/belief in COVID-19. The third component supported previous observations that greater exposure to the practical implications of COVID pandemic constitutes a separable predictor for people’s coping strategies and compliance with recommendations of the local health authorities (Fluharty & Fancourt, 2020; Wright et al., 2020).

**Table 2.** Mean loadings of the principal component analysis of questionnaire responses.

Question	Component		
	1	2	3
Conservative political orientation (SI Q20)	**0.72**	**−0.37**	0.05
Doubt in the danger of COVID-19 (SI Q53)	**0.68**	**−0.37**	−0.06
Doubt in the usefulness of face masks against COVID-19 (SI Q54)	**0.64**	−0.30	−0.07
Political orientation of friends & family (SI Q21)	**0.57**	−0.20	−0.02
Age (SI Q2)	**0.56**	0.16	**0.44**
Do you tend to be an anxious person? (reversed, SI Q16)	**0.52**	−0.10	−0.04
Do you identify with a religion? (reversed, SI Q12)	**−0.42**	0.14	0.05
Frequency of checking news unrelated to COVID (SI Q23)	0.27	**0.78**	0.00
Interest in politics (SI Q18)	0.13	**0.76**	−0.04
Frequency of news checking before COVID-19 (SI Q24)	0.35	**0.70**	0.01
Frequency of COVID news checking (SI Q22)	0.25	**0.70**	0.04
Do you enjoy debating political issues? (SI Q19)	0.15	**0.54**	−0.18
Frequency of meeting people offline on a typical week (SI Q52)	0.28	0.11	**−0.73**
Frequency of meeting people offline during past week (SI Q51)	0.25	0.11	**−0.69**
Is a mask mandatory for your work? (reversed, SI Q50)	0.01	−0.03	**0.56**
Are you a student? (reversed, SI Q10)	0.38	0.03	**0.42**
Vulnerability to COVID-19? (SI Q26)	−0.09	0.33	**0.36**
Are you working from home? (SI Q49)	−0.03	0.04	**0.27**

The strongest seven loadings for each component are shown in bold font. See Supplementary materials for the list of all 26 questions and their loadings. Question numbers refer to items listed in the Supplementary materials.

Each participant was assigned a score based on their average factor loading for these three components. These scores were then used to predict attractiveness and intensity ratings of the images, along with the frequency and duration of wearing protective masks. For ease of interpretation, we have reversed the sign of components 1 and 3, so that higher scores correspond to greater belief in the danger of COVID on component 1, and with greater exposure to COVID on component 3.

### Individual differences in mask attractiveness predicted by frequency of use and PCA factors

In all, 379 participants responded to all survey questions and completed the rating task. We used simultaneous multiple linear regression to predict the attractiveness of protective masks for these participants using five main predictor variables. Two of the variables concerned frequency of mask wearing, which had three levels (rarely, sometimes, very often). These levels were dummy-coded to allow a comparison between occasional mask wearers (rarely vs. sometimes) and frequent mask wearers (rarely vs. often). The other three variables were participants’ factor loadings on the three PCA components (belief in the danger of COVID-19, interest in politics, and situational exposure to COVID-19). A sixth covariate was added to the model (attractiveness ratings of the recreational masks) to control for a participant’s baseline tendency to provide generally low or high ratings. To anticipate the results, including or removing this predictor had no influence on the pattern or the significance of the results.

**Figure 4.**
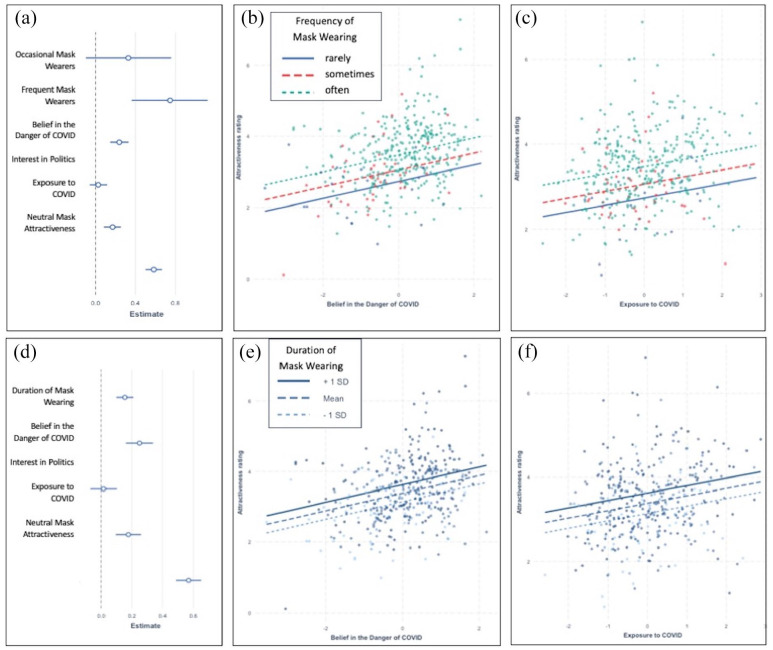
A summary of the regression predicting attractiveness of protective masks from beliefs in the danger of COVID, interest in politics, exposure to COVID, frequency (a to c) or duration (d to f) of mask wearing, and attractiveness of neutral masks. Panel (a) shows regression coefficients for the model that includes frequency of use, with predictors shown on the vertical axis, and their respective betas on the horizontal axis. The lines represent the 95% CI of the betas. Panel (d) shows the respective regression coefficients for the model with duration of use. Panels (b, c), (e, f) show the effects of frequency or duration of mask wearing, respectively, and belief in the danger of COVID (b, e) or exposure to COVID (c, f) on attractiveness ratings. The dots represent partial residuals. For zero-order correlations between all variables see Table SI3.

The overall model fit was *R*^2^ = .402. Figure 4a shows the standardised regression coefficients for the five predictors and the covariate. Three of the four predictors were significantly associated with attractiveness ratings for protective masks; only interest in politics was not, *p* > .5. Higher attractiveness ratings were associated with a stronger belief in the danger of COVID, β = .24, *t*(372) = 4.98, *p* < .001, with greater environmental exposure to COVID, β = .17, *t*(372) = 3.9, *p* < .001, and with more frequent use of protective masks, β_*never_vs_sometimes*_ = .33, β_*sometimes_vs_often*_ = .74, *F*(2, 372) = 10, *p* < .001. Figure 4b and c shows that even after accounting for belief in the danger of COVID and exposure to COVID, more frequent mask wearing was still significantly associated with higher attractiveness ratings. This result is consistent with the proposal that simply using a mask in daily life is associated with its positive emotional appraisal, since the correlation between frequency of use and appraisal held up even after controlling for personally held ideological beliefs (belief in the danger of COVID) and controlling for external environmental circumstances (exposure to COVID).

A test of the specificity of this interpretation was obtained by conducting the same simultaneous multiple regression analysis on attractiveness ratings for the COVID-unrelated masks (ski and diving masks). The overall model fit was very poor, *R*^2^ = .069. Neutral mask attractiveness ratings were negatively associated with belief in the danger of COVID, β = −.29, *t*(373) = −5.2, *p* < .001, and negatively associated with interest in politics, β = −.13, *t*(373) = −2.34, *p* = .0198, all other *ps* > .17. This means that participants with a stronger belief in the danger of COVID tended to rate recreational masks as less attractive, and so did participants who had greater interest in politics. Neither exposure to COVID, nor frequency of protective mask wearing correlated significantly with the attractiveness of recreational masks. This analysis confirms that the associations observed between mask attractiveness, frequency of mask wearing, and exposure to COVID were specific to protective masks. Most importantly, the association observed between the attractiveness of masks and belief in the danger of COVID ran in the opposite direction for protective and recreational masks: Belief in the danger of COVID was positively associated with the attractiveness of protective masks but negatively associated with the attractiveness of recreational masks. It suggests that participants who were suspicious of the dangers of COVID found protective masks less attractive and, when they were among the stimuli, may have compensated by rating recreational masks as more attractive.

### Individual differences in mask attractiveness predicted by duration of mask use and PCA factors

We conducted the same set of simultaneous multiple regressions to predict protective mask attractiveness after replacing the frequency of mask use with the duration of mask wearing (coded as an interval variable). Figure 4d shows the standardised regression coefficients for the five predictors and shows the same patterns as revealed in the previous analysis of all the predictors, only interest in politics was not associated with perceived attractiveness of the masks. The overall model fit was *R*^2^ = .42. Attractiveness ratings for protective masks was associated significantly with belief in the danger of COVID, β = .25, *t*(373) = 5.49, *p* < .001, exposure to COVID, β = .18, *t*(373) = 4.16, *p* < .001, and duration of mask wearing, β = .15, *t*(373) = 5.35, *p* < .001, predicted higher ratings, while interest in politics did not, *p* > .7. As shown in Figure 4, panels e and f, and converging with the results reported above, duration of mask wearing was associated with higher attractiveness ratings for protective masks even when other attitudes and situational factors were controlled for.

A parallel test of specificity on attractiveness ratings of COVID-unrelated masks indicated a weak overall model fit, *R*^2^ = .085. Belief in the danger of COVID and interest in politics were both negatively associated with attractiveness ratings, β = −.3, *t*(374) = −5.6, *p* < .001 and *β* = −.14, *t*(374) = −2.68, *p* = .008, respectively. In addition, duration of mask wearing was positively associated with attractiveness ratings for diving and ski masks, β = .097, *t*(374) = 2.8, *p* = .005. Thus, the longer people were wearing protective masks, the higher their ratings were for both protective and recreational masks. Yet the association between ratings of protective masks and duration of their use survived controlling for the ratings of recreational masks, as well as the other factors related to individual differences.

### Individual differences in appraisal of masks: intensity ratings

**Figure 5.**
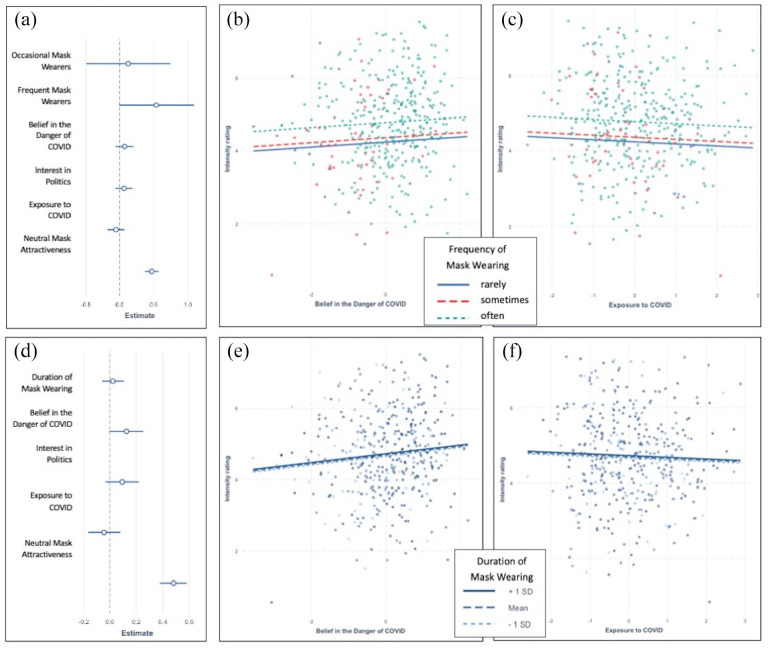
A summary of the regression predicting emotional intensity evoked by protective masks from beliefs in the danger of COVID, interest in politics, exposure to COVID, frequency (a to c) or duration (d to f) of mask wearing, and intensity ratings for neutral masks. Panel (a) shows regression coefficients for the model that includes frequency of use, with predictors shown on the vertical axis, and their respective betas on the horizontal axis. The lines represent the 95% CI of the betas. Panel (d) shows the respective regression coefficients for the model with duration of use. Panels (b, c), (e, f) show the effects of frequency or duration of mask wearing, respectively, and belief in the danger of COVID (b, e) or exposure to COVID (c, f) on intensity ratings. The dots represent partial residuals. For zero-order correlations between all variables, see Table SI4.

We used the same analytic approach for the emotional intensity ratings as already reported for attractiveness ratings: A simultaneous multiple regression predicting intensity of emotions evoked by protective masks from frequency of mask wearing (dummy coded), the three PCA components, and intensity of emotions for recreational masks (see Figure 5). There was a significant association with frequency of wearing protective masks, β_*never_vs_sometimes*_ = .12, β_*sometimes_vs_often*_ = .54, *F*(2, 372) = 3.37, *p* = .035. None of the PCA components were significant, *ps* > .3. The overall model fit was *R*^2^ = .1996.

On the parallel test of specificity for recreational masks, the overall model fit was poorer, *R*^2^ = .038. Ratings for recreational masks were negatively associated with belief in the danger of covid, β = −.28, *t*(373) = −4.09, *p* < .001. No other variables had a significant effect, *ps* > .09. This pattern of results repeats the pattern observed for attractiveness ratings; frequency of mask wearing was associated with greater intensity of emotions evoked by protective, but not recreational masks. And this association held up even after other attitudinal and situational factors are controlled for.

The analysis testing the duration of mask wearing (rather than frequency) showed only a few significant associations for either protective or recreational masks. For protective masks, the model fit was *R*^2^ = .188, and the only notable effect was a trend for association with belief in the danger of COVID, β = .12, *t*(373) = 1.9, *p* = .058. No other effects were significant, *ps* > .15. For recreational masks, the model fit was poorer, *R*^2^ = .0397, with the only significant association emerging with beliefs in the danger of COVID, β = −.26, *t*(374) = −3.99, *p* < .001.

In summary, the rated intensity of emotions evoked by the protective masks was not reliably predicted by either of the three PCA components. However, once these three factors were controlled, there was a moderate association between intensity and frequency of use, but not between intensity and duration of mask wearing.

### Within-participant replication

In October 2020, we contacted participants who had participated in our study in July-August, asking them to do exactly the same tasks and answer the same survey. Our initial aim had been to investigate whether new users—that is, people who picked up mask-wearing practices between the two waves of data collection—would show the hypothesised effects of practice on emotional appraisal. However, given the high rates of mask use already in July–August 2020, we did not expect to see much change in mask-wearing practices (this was indeed the case). Yet we still could investigate whether two additional months of mask-wearing resulted in higher emotional ratings. To this end, we conducted a repeated measures ANOVA with time of testing (July–August vs. October) as a within-participant variable. In addition, we tested reliability of the results obtained in the first wave of testing.

Participants were contacted through Prolific; 330 of the original participants consented to the re-testing and completed the follow-up study, and 326 of them completed all the tasks and answered all the questions. This sub-sample was balanced in gender (44.6% male, 53.7% female participants) and ranged in age from 18 to 78 (mean 36.2, median 33). 38 participants (11.3%) had been diagnosed with COVID-19 or suspected they had it.

**Figure 6.**
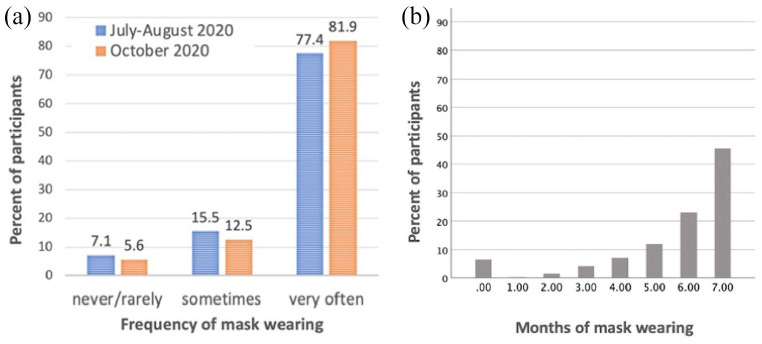
Frequency and duration of wearing of protective masks by study participants in October 2020. Panel (a) frequency of mask wearing in the first and second wave of data collection. Panel (b) duration of mask wearing in the second wave of data collection.

In fall (October 2020), more participants were wearing masks on a regular basis (very often), $\chi^{2}$ (4) = 152, *p* < .001, see also Figure 6. However, as overwhelming majority of participants had been wearing masks very often already in the summer (July–August 2020), the number of people who reported changing their habits was not enough to test the effects of changes in mask wearing practices on anything else.

### The effect of frequency of mask wearing in the second wave

We compared participants’ ratings for protective and recreational masks between the first (July-August, 2020) and the second (October, 2020) wave of data collection. Our primary question was whether the effects we had seen for frequency of mask wearing would replicate in a second occasion of testing. Hoping to replicate the initial finding, we used exactly the same stimuli in the second wave of testing. But doing this introduced the possibility that participants would be habituated to the specific images they had rated earlier. A secondary question was therefore whether testing the same images only a few months later would have an additional effect. To answer these two questions, we included frequency of mask-wearing (as of October 2020) as a between participant factor, resulting in a 2 (time: July–August vs. October, within-participant) × 2 (mask type: protective vs. recreational, within-participant) × 3 (Frequency of mask wearing: rarely vs. sometimes vs. often, between participants) mixed model ANOVA.

**Figure 7.**
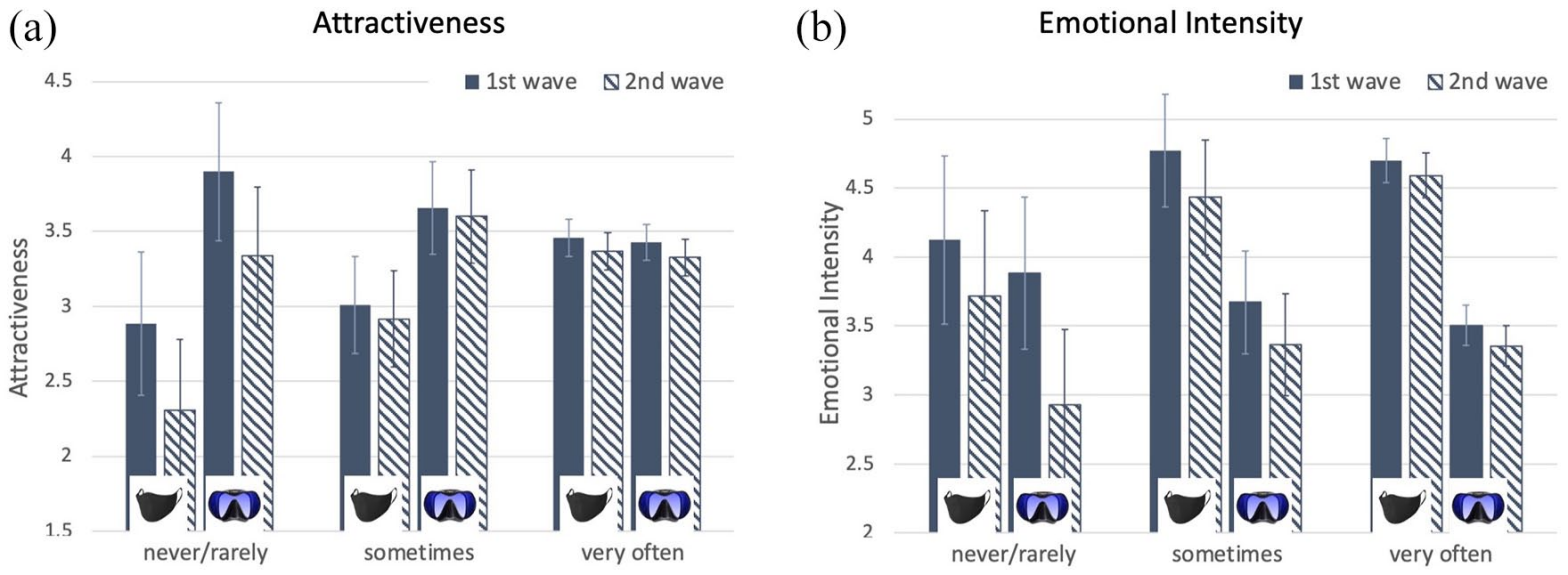
Mean rating of attractiveness (panel a) and emotional intensity (panel b) on a 7-point scale for protective and recreational masks for the first wave of testing (solid colour) and second wave of testing (striped) as a function of frequency of mask wearing. Error bars show 95% confidence intervals.

Figure 7a shows the results for attractiveness ratings. Our primary finding was that attractiveness ratings were higher for protective masks when participants wore them more frequently (*M_frequent_* = 3.73, *M_sometimes_* = 3.28, *M_infrequent_* = 2.91, *F*(2, 335) = 9.20, *p* < .001, $\eta_{p}^{2}=.052$ . This simple effect for protective masks was stronger at time 2 than time 1, supported by an interaction between frequency and time for protective masks, *F*(2, 335) = 3.47, *p* = .0321, $\eta_{p}^{2}=.02$ . When both protective and recreational masks were included in the analysis, the two-way interaction of frequency of mask use × mask type was significant, *F*(2, 335) = 18.87, *p* < .001, $\eta_{p}^{2}=.101$ , reflecting a positive association between ratings and frequency of use for protective masks and a negative association for recreational masks. The three-way interaction with time was not significant, *F* < 1, *p* > .9, indicating that this pattern was stable across time of testing.

The secondary finding in these data was an overall decrease in attractiveness for both protective and recreational images from time 1 to time 2, *F*(1, 335) = 14.6, *p* < .001, $\eta_{p}^{2}=.042$ . This decrease was larger for infrequent mask-wearers than for those who wear the protective masks sometimes or very often, *F*(2, 335) = 4.42, *p* = .013, $\eta_{p}^{2}=.026$ . There was no interaction between time of testing and mask type, *F* < 1, *p* > .8.

Figure 7b shows that a similar pattern of results held for the emotional intensity ratings. These ratings for protective masks were also higher for more frequent mask users at both times 1 and 2, *F*(1, 335) = 6.44, *p* = .011, $\eta_{p}^{2}=.019,$ but with no interaction with time of testing, *p* > .3. Again, for recreational masks ratings were lower with greater frequency of use, as evidenced by an interaction between mask type and frequency of mask use, *F*(2, 335) = 2.92, *p* = .056, $\eta_{p}^{2}=.017$ .

The secondary effect of time of testing was also similar to that observed for the attractiveness ratings. Emotional intensity ratings decreased from the first to the second time of testing, *F*(1, 335) = 16.8, *p* < .001, $\eta_{p}^{2}=.048$ , for protective and recreational masks alike, interaction *p* > .12. The decrease over time was greater for infrequent mask-wearers than for participants who wore them often, *F*(2, 335) = 3.325, *p* = .037, $\eta_{p}^{2}=.019$ .

In summary, the association between frequency of mask wearing and rated attractiveness we observed at time 1 was even stronger when we tested it at time 2. The association between emotional intensity and frequency of mask wearing was also stable over time. We interpret these two findings to imply that protective masks were more attractive and more emotionally engaging for wearers who used them more frequently. A secondary finding was that ratings for product images were generally lower at time 2 than at time 1. This finding was not specific to protective masks, but applied equally to both protective and recreational masks. We interpret this finding as stemming from repeated viewing of the same test images, rather than being related to the wearing of protective masks. This is consistent with numerous previous reports of a decrease in emotional appeal when the same stimulus is tested repeatedly over time (Berlyne, 1970; Biederman & Vessel, 2006; Isik & Vessel, 2019). The decreased appeal of repeated stimulation is interpreted in that literature as an effect of habituation, likely stemming from reward circuits in the brain responding less vigorously to stimuli that are recognised as identical to ones seen previously. In any case, the data are clear in pointing to the independence of the specific effects of wearing protective masks from the more general effects of being repeatedly tested on the same stimuli.

The significant interaction of frequency of mask wearing × time, seen for both attractiveness ratings and emotional intensity ratings, were largely due to the small number of participants (*n* = 19) in the never/rarely group. Ratings overall in this group decreased more than in any other group from time 1 to time 2. When we examined other data from this group more closely, we noted that they tended to be older than our sample as a whole (mean age = 42.7 vs. 35.8 years), disproportionately male (63% vs. 37%), and from regions that were relatively rural and with lower rates of mask wearing (e.g., Alaska, Alberta, Arizona, Missouri, North Dakota). It is difficult to know whether to attribute this group’s reduced ratings over time to measurement error (because of the small sample size) or to increased polarisation of opinions over time among infrequent mask wearers, but their data do contribute to even stronger associations between mask wearing and the emotional appeal of protective mask at time 2 than at time 1.

### Individual differences in mask appraisal on the second wave

To re-test the results regarding individual differences in mask appraisal, we first conducted a confirmatory PCA on the survey questions. We then used the newly calculated component scores to predict emotional ratings for masks, as of October 2020, from mask wearing practices (frequency / duration) and the three PCA components. To anticipate the results, the main pattern of findings reported earlier was replicated in all important respects.

Confirmatory PCA was conducted using *lavaan* package for *R* (Rosseel, 2012). The model fit was satisfactory, albeit not perfect, adjusted goodness of fit (AGFI) = .75, *RMSEA* = .09, *SRMR* = .101, *CFI* = .674, $\chi^{2}$ (298) = 1,090.7, *p* < .001. Component loadings are presented in Supplementary Table SI2. We used component scores from this model and frequency/duration of mask use to predict emotional ratings, testing the same models as described earlier.

The multiple regression predicting attractiveness of protective masks from frequency of mask wearing (dummy coded), the three PCA components, and attractiveness of recreational masks had an overall model fit of *R*^2^ = .272. It revealed a significant effect of frequency of mask wearing, β_*never_vs_sometimes*_ = .19, β_*sometimes_vs_often*_ = .64, *F*(2, 319) = 10.8, *p* = .002, and belief in the danger of COVID, β = .32, *t*(319) = 5.17, *p* < .001. There was also a trend for a negative association with interest in politics, β = −.103, *t*(319) = −1.86, *p* = .064, all other *ps* > .27. The test of specificity involving recreational masks yielded only a main effect of beliefs in the danger of COVID emerged, β = −.24, *t*(320) = −3.65, *p* < .001, for frequency of mask wearing *ps* > .19, all other *ps* > .07. The overall fit was *R*^2^ = .041

The overall pattern therefore closely resembled the data collected originally. The attractiveness of protective masks was associated with frequency of mask wearing above and beyond its association with beliefs in the danger of COVID. The specificity of this finding was again evident in that the attractiveness of recreational masks was not associated with frequency of mask-wearing, and its association with beliefs in the danger of COVID was negative.

One subtle difference we observed between the two occasions was that situational exposure to COVID no longer accounted for any significant variance in the attractiveness of protective masks. While these observational data do not warrant strong causal interpretations, this finding is consistent with personal experience shaping one’s emotional appraisal of an object. Note that in the summer (July–August 2020—first wave of data collection), situational exposure forced some people to adopt mask-wearing sooner than others, thus amplifying the difference between people who had to use the masks regularly and those who could choose whether to do so or not. By October 2020 (the replication), even infrequent mask-wearers had become accustomed to seeing masks on others and being required to wear masks in some situations.

The multiple regression predicting attractiveness of protective masks based on duration of mask-wearing (interval, in months) rather than frequency had a model fit of *R*^2^ = .258. Significant predictors included duration of mask wearing, β = .069, *t*(320) = 2.29, *p* = .023, and belief in the danger of COVID, β = .33, *t*(320) = 5.17, *p* < .001. There was once again a trend for an association with interest in politics, β = −.109, *t*(320) = −1.95, *p* = .052, all other *ps* > .2.

The multiple regression predicting attractiveness of recreational masks had a very poor overall fit, *R*^2^ = .077. Yet, here was a significant association with duration of mask wearing, β = .11, *t*(321) = 3.6, *p* < .001, along with a negative effect of belief in the danger of COVID, β = −.308, *t*(321) = −4.78, *p* < .001, all other *ps* > .09. We found it surprising that the attractiveness of recreational masks should increase with a longer duration of wearing protective masks, but as seen in the previous analyses, attractiveness of protective masks was predicted by duration of mask wearing even when the attractiveness of recreational masks was statistically controlled. In general, the pattern closely resembled that obtained in the first wave of the study.

The emotional intensity of protective masks was predicted only by interest in politics, β = .21, *t*(319) = 2.86, *p* = .004, but not by frequency of mask wearing, *F*(2, 319) = 1.5, *p* = .2, overall fit *R*^2^ = .176. In a parallel model, duration of mask wearing did not have any effect either, *p* = .08, model fit *R*^2^ = .178. We interpret these finding to imply that the effect of wearing protective masks on the intensity of emotions was eliminated by October 2020.

To summarise, a within-participant replication of the study revealed that by October 2020, a larger number of people were wearing masks more frequently than in July–August 2020. Consistent with this observation of a significant change in baseline behaviour, the effects of mask-wearing frequency and duration had disappeared from ratings of the intensity of emotion elicited by the images, although they were still clearly evident in the ratings of attractiveness.

## Discussion

The purpose of this study was to find ecological support for the well-established laboratory finding that merely acting on or using a novel object can increase its emotional appeal (Fenske & Raymond, 2006; Hayes et al., 2008; Peck & Shu, 2009; Schonberg et al., 2014; Streicher & Estes, 2015; Wispinski et al., 2020). This process, however, is potentially attenuated with prolonged use, as humans tend to show decline in preferences for familiar objects (Biederman & Vessel, 2006; Isik & Vessel, 2019), making ecological studies on the relationship between use and emotional appraisal challenging. The circumstances of the growing COVID-19 pandemic, in spring and summer of 2020, offered us a unique opportunity to overcome this challenge. The pandemic introduced novel objects into our daily lives, namely protective masks and other safety-related objects, with which a majority of people had little or no previous experience. Moreover, as the pandemic unfolded, it provided an opportunity to study the association between object usage and emotional appraisal separately in people who began using these objects earlier versus later, and had different reasons for doing so, including reasons linked to personal ideology and reasons linked to situational circumstances.

As we noted in the “Introduction” section, an observational study design like this one lacks many of the controls that are possible in a laboratory study. But in our view, the bi-directional causality seen in laboratory studies is no longer in question. Instead, an important unanswered question is whether these factors are operational in the everyday world as people quite naturally come into contact with novel objects and change their patterns of use with respect to them. Studying the emotional appraisal of protective face masks, which suddenly grew in popularity world-wide during 2020, therefore offered a ready-made opportunity to answer this question.

For comparison stimuli we chose a variety of products that were similar to protective masks in several respects but differed from them in others. COVID-unrelated face masks that are used in recreation offered one such comparison (e.g., ski and diving masks). COVID-related products that were not as novel as masks offered another (e.g., latex gloves, personal hygiene products). Our understanding of the laboratory evidence led us to expect that the association between use and emotional appraisal would be more pronounced for the objects made novel by the pandemic (i.e., protective masks) than for other objects that were previously familiar and/or used more frequently.

One unanticipated benefit of the pandemic for our research was the rapid pivot among cognitive researchers to testing people online rather than in the laboratory. The rapid development of software and research approaches to facilitate remote testing, along with public acceptance of remote research as the “new-normal,” meant that we were able to access a much more diverse set of participants than is typical in laboratory studies.

The main findings of the study supported the *usage-positive appraisal hypothesis*. The results clearly indicated that both the frequency of wearing a mask, and the duration of wearing it, were linked to the positive emotional appraisals of these objects. This interpretation is supported by the theory of microvalences (Manaligod, 2020; Lebrecht et al., 2012; Lebrecht & Tarr, 2012), which proposes that everyday objects are implicitly associated with low levels of positive and negative valence that influence visual processing (Lebrecht et al., 2011) and inform choices (Lebrecht & Tarr, 2010). Unlike more strongly-valenced responses to stimuli that universally signal threat or reward, microvalences emerge in the course of everyday life as a consequence of our encounters with objects, and are thus particular to an individual’s experience. This phenomenon has been demonstrated for consumer choices and preferences measured and manipulated in the lab (Lebrecht et al., 2011; Lebrecht & Tarr, 2010; Manaligod, 2020). The present study is the first to demonstrate experience-based positive microvalence to be associated with common real-world experiences with specific categories of objects.

There were also important secondary lines of evidence in this study that bolstered this interpretation. For instance, when we examined the data for latex gloves (covid-related, familiar, but infrequently used in the past) the data showed it was more similar to the data for protective masks than it was for personal hygiene products (familiar and frequently used by many previously). This interpretation was strengthened even further when we distinguished between participants who made regular use of these products pre-pandemic and those who did not. Those for whom these products were relatively more novel provided data that was more similar to that for protective masks.

But perhaps the strongest evidence supporting a causal arrow linking the usage of protective masks with their positive emotional appraisal came when we compared the relative contributions of personal ideology (doubt/belief in the dangers of covid), environmental circumstances (workplace requirements to wear masks), and frequency of mask usage to ratings of mask attractiveness. Even though the expected links between doubt/belief in the dangers of covid and mask attractiveness were evident in the data (i.e., masks are less attractive to those who doubt the danger), and even though the expected links between workplace requirements and mask attractiveness were in evidence (i.e., masks were more attractive if they were a requirement of work), there was still a significant unique contribution to mask attractiveness that could be attributed to the frequency and duration of wearing a mask.

The literature on perception and emotion teaches us that mere exposure to an object (Gordon & Holyoak, 1983; Griffiths & Mitchell, 2008; Reber et al., 2004; Zajonc, 1968) can lead to more positive appraisal in the lab. This finding appears to be dependent, or at a minimum is heightened further, when that exposure to the object occurs in a positive context (Hayes et al., 2008; Roos et al., 2013; Schonberg et al., 2014). Is it possible that the present results represent a naturalistic case of the mere exposure effect? According to the mere exposure hypothesis, simply encountering novel protective masks should have increased their emotional appeal. And with the onset of the pandemic, most people were exposed to protective masks in everyday usage. We do not favour this interpretation, because it is not consistent with the large individual differences in attractiveness we observed. Yet, a critic might argue, what about the possibility that some people rarely saw masks because the tended to stay at home, and that it is this variable that contributes to the large individual differences? We do not favour this interpretation either because, regardless of their degree of public interaction, all participants were exposed to the sight of protective masks to a much greater extent than they had seen prior to the pandemic. That factor alone should have made them more attractive in general. The present findings that personal mask usage has an association with their emotional appeal goes beyond the mere exposure hypothesis because it suggests that one’s actions—over and above one’s perception—is associated with an object’s attractiveness. We acknowledge that future research will be needed to fully tease apart the relative strength of the contributions to attractiveness that arise from merely seeing an object, versus attending to or making a decision with respect to a seen object, versus physically handling the object. In this context, the present data should be seen as a first step in showing that usage in the everyday world is associated with an object’s emotional appeal.

An alternative account of these findings is that people who adopted protective masks early were those who initially held a more positive appraisal of them. Indeed, multiple studies show that a positive appraisal increases the tendency to approach an object (Chen & Bargh, 1999; Lewin, 1935; Phaf et al., 2014). While this is indeed a possible contributing factor, our finding that the duration of experience with protective masks is directly linked to participants’ appraisal of them suggests that there is a dynamic reinforcing relationship between emotional appraisal and behavioural adoption.

An encouraging practical application of the present data, in conjunction with the theory of microvalence, is that taking societal measures to encourage mask wearing when there is a pandemic may help to move their emotional appraisal from negative to positive. It should give us hope that, among frequent and long-time wearers of protective masks, the emotional appraisal given to COVID-19 masks rivals those given to recreational and personal hygiene products.

## Supplemental Material

sj-docx-1-qjp-10.1177_17470218211037128 – Supplemental material for In the hands of the beholder: Wearing a COVID-19 mask is associated with its attractiveness Supplemental material, sj-docx-1-qjp-10.1177_17470218211037128 for In the hands of the beholder: Wearing a COVID-19 mask is associated with its attractiveness by Veronica Dudarev, Maria GM Manaligod, James T Enns and Rebecca M Todd in Quarterly Journal of Experimental Psychology

sj-docx-2-qjp-10.1177_17470218211037128 – Supplemental material for In the hands of the beholder: Wearing a COVID-19 mask is associated with its attractiveness Supplemental material, sj-docx-2-qjp-10.1177_17470218211037128 for In the hands of the beholder: Wearing a COVID-19 mask is associated with its attractiveness by Veronica Dudarev, Maria GM Manaligod, James T Enns and Rebecca M Todd in Quarterly Journal of Experimental Psychology

sj-docx-3-qjp-10.1177_17470218211037128 – Supplemental material for In the hands of the beholder: Wearing a COVID-19 mask is associated with its attractiveness Supplemental material, sj-docx-3-qjp-10.1177_17470218211037128 for In the hands of the beholder: Wearing a COVID-19 mask is associated with its attractiveness by Veronica Dudarev, Maria GM Manaligod, James T Enns and Rebecca M Todd in Quarterly Journal of Experimental Psychology

sj-jpeg-4-qjp-10.1177_17470218211037128 – Supplemental material for In the hands of the beholder: Wearing a COVID-19 mask is associated with its attractiveness Supplemental material, sj-jpeg-4-qjp-10.1177_17470218211037128 for In the hands of the beholder: Wearing a COVID-19 mask is associated with its attractiveness by Veronica Dudarev, Maria GM Manaligod, James T Enns and Rebecca M Todd in Quarterly Journal of Experimental Psychology

## Notes

1. A more deliberate approach would suggest excluding any demographic information from the PCA, as participant demographic and socioeconomic characteristics could be correlated with or predict the latent constructs such as belief in the danger of COVID, but not be caused by them. Contrary to that, responses to self-report questions about attitudes and behaviours could be influenced by the latent constructs. Restricting PCA to only questions about beliefs and behaviours revealed a very similar pattern and made the correlations with ratings of masks even stronger.

## References

[bibr1-17470218211037128] Anderson B. A. (2016). The attention habit: How reward learning shapes attentional selection. Annals of the New York Academy of Sciences, 1369(1), 24–39. 26595376 10.1111/nyas.12957

[bibr2-17470218211037128] Aratani L. (2020, June 29). How did face masks become a political issue in America? The Guardian. https://www.theguardian.com/world/2020/jun/29/face-masks-us-politics-coronavirus

[bibr3-17470218211037128] Beer T. (2020, July 16). Anti-mask rallies continue in U.S. amid rising coronavirus cases and deaths. Forbes. https://www.forbes.com/sites/tommybeer/2020/07/16/anti-mask-rallies-continue-in-us-amid-rising-coronavirus-cases-and-deaths/#708d50d52246

[bibr4-17470218211037128] Berlyne D. E. (1970). Novelty, complexity, and hedonic value. Perception & Psychophysics, 8(5), 279–286.

[bibr5-17470218211037128] Biederman I. Vessel E. (2006). Perceptual pleasure and the brain: A novel theory explains why the brain craves information and seeks it through the senses. American Scientist, 94(3), 247–253.

[bibr6-17470218211037128] Chelazzi L. Perlato A. Santandrea E. Della Libera C. (2013). Rewards teach visual selective attention. Vision Research, 85, 58–72. 23262054 10.1016/j.visres.2012.12.005

[bibr7-17470218211037128] Chen M. Bargh J. A. (1999). Consequences of automatic evaluation: Immediate behavioral predispositions to approach or avoid the stimulus. Personality and Social Psychology Bulletin, 25(2), 215–224.

[bibr8-17470218211037128] De Vito D. Fenske M. J . (2018). Affective evidence that inhibition is involved in separating accessory representations from active representations in visual working memory. Visual Cognition, 26(8), 583–600.

[bibr9-17470218211037128] Failing M. Theeuwes J. (2018). Selection history: How reward modulates selectivity of visual attention. Psychonomic Bulletin & Review, 25(2), 514–538. 28986770 10.3758/s13423-017-1380-y PMC5902518

[bibr10-17470218211037128] Fenske M. J. Raymond J. E. (2006). Affective influences of selective attention. Current Directions in Psychological Science, 15(6), 312–316.

[bibr11-17470218211037128] Fenske M. J. Raymond J. E. Kunar M. A. (2004). The affective consequences of visual attention in preview search. Psychonomic Bulletin & Review, 11(6), 1055–1061. 15875975 10.3758/bf03196736

[bibr12-17470218211037128] Fluharty M. Fancourt D . (2020). How have people been coping during the COVID-19 pandemic? Patterns and predictors of coping strategies amongst 26,580 UK adults. 10.31234/osf.io/nx7y5 PMC8280648 34266498

[bibr13-17470218211037128] Gandhi M. Beyrer C. Goosby E. (2020). Masks do more than protect others during COVID-19: Reducing the inoculum of SARS-CoV-2 to protect the wearer. Journal of General Internal Medicine, 35(10), 3063–3066. 32737790 10.1007/s11606-020-06067-8 PMC7393808

[bibr14-17470218211037128] Gordon P. C. Holyoak K. J. (1983). Implicit learning and generalization of the “mere exposure” effect. Journal of Personality and Social Psychology, 45(3), 492–500.

[bibr15-17470218211037128] Griffiths O. Mitchell C. J. (2008). Negative priming reduces affective ratings. Cognition and Emotion, 22(6), 1119–1129.

[bibr16-17470218211037128] Hayes A. E. Paul M. A. Beuger B. Tipper S. P. (2008). Self produced and observed actions influence emotion: The roles of action fluency and eye gaze. Psychological Research, 72(4), 461–472. 17899177 10.1007/s00426-007-0125-3

[bibr17-17470218211037128] Isik A. I. Vessel E. A. (2019). Continuous ratings of movie watching reveal idiosyncratic dynamics of aesthetic enjoyment. PLOS ONE, 14(10), Article e0223896. 10.1371/journal.pone.0223896 PMC6814238 31652277

[bibr18-17470218211037128] Kang M. J. Hsu M. Krajbich I. M. Loewenstein G. Mcclure S. M. Wang J. T. Camerer C. F. (2014). The hunger for knowledge: Neural correlates of curiosity. Psychological Science, 20(8), 1–20. 10.1111/j.1467-9280.2009.02402.x 19619181

[bibr19-17470218211037128] Karalis K. (2020, September 14). No more masks: Hundreds attend anti-mask mandate rally in St. George. ABC4. https://www.abc4.com/news/top-stories/no-more-masks-hundreds-attend-anti-mask-mandate-rally-in-st-george/

[bibr20-17470218211037128] Kiss M. Goolsby B. A. Raymond J. E. Shapiro K. L. Silvert L. Nobre A. C. … Eimer M. (2007). Efficient attentional selection predicts distractor devaluation: Event-related potential evidence for a direct link between attention and emotion. Journal of Cognitive Neuroscience, 19(8), 1316–1322. 17651005 10.1162/jocn.2007.19.8.1316 PMC2397542

[bibr21-17470218211037128] Knotek E. S. Schoenle R. S. Dietrich A. M. Müller G. J. Myrseth K. O. R. Weber M. (2020, July 16). Consumers and COVID-19: Survey results on mask-wearing behaviors and beliefs. Economic Commentary. https://www.clevelandfed.org/newsroom-and-events/publications/economic-commentary/2020-economic-commentaries/ec-202020-survey-results-on-mask-wearing-behaviors-and-beliefs.aspx

[bibr22-17470218211037128] Lebrecht S. Bar M. Barrett L. F. Tarr M. J. (2012). Micro-valences: Perceiving affective valence in everyday objects. Frontiers in Psychology, 3, Article 107. 10.3389/fpsyg.2012.00107 PMC3328080 22529828

[bibr23-17470218211037128] Lebrecht S. Bar M. Sheinberg D. L. Tarr M. J. (2011). Micro-valence: Nominally neutral visual objects have affective valence. Journal of Vision, 11(11), 856.

[bibr24-17470218211037128] Lebrecht S. Tarr M. (2010). Defining an object’s micro-valence through implicit measures. Journal of Vision, 10(7), 966–966.

[bibr25-17470218211037128] Lebrecht S. Tarr M. J. (2012). Can neural signals for visual preferences predict real-world choices? BioScience, 62(11), 937–938.

[bibr26-17470218211037128] Lewin K. (1935). A dynamic theory of personality. McGraw Hill.

[bibr27-17470218211037128] Lyu W. Wehby G. L. (2020). Community use of face masks and COVID-19: Evidence from a natural experiment of state mandates in the US. Health Affairs, 39(8), 1419–1425. 32543923 10.1377/hlthaff.2020.00818

[bibr28-17470218211037128] Manaligod M. G. M. (2020). Attentional biases by induced microvalence in novel objects: An emphasis on the role of experience. University of British Columbia.

[bibr29-17470218211037128] Miller A. M. Brueck H. (2020, July 28). Anti-maskers are the new anti-vaxxers. Business Insider. https://www.businessinsider.com/anti-maskers-are-new-anti-vaxxers-threatening-public-health-coronavirus-2020-7

[bibr30-17470218211037128] Peck J. Shu S. B. (2009). The effect of mere touch on perceived ownership. Journal of Consumer Research, 36(3), 434–447.

[bibr31-17470218211037128] Phaf R. H. Mohr S. E. Rotteveel M. Wicherts J. M. (2014). Approach, avoidance, and affect: A meta-analysis of approach-avoidance tendencies in manual reaction time tasks. Frontiers in Psychology, 5, Article 378. 10.3389/fpsyg.2014.00378 PMC4021119 24847292

[bibr32-17470218211037128] Pierce J. W. Gray J. R. Simpson S. MacAskill M. R. Hochenberger R. Sogo H. … Lindelov J. (2019). PsychoPy2: Experiments in behavior made easy. Behavior Research Methods, 51, 195–203. 30734206 10.3758/s13428-018-01193-y PMC6420413

[bibr33-17470218211037128] Raymond J. E. Fenske M. J. Westoby N. (2005). Emotional devaluation of distracting patterns and faces: A consequence of attentional inhibition during visual search? Journal of Experimental Psychology: Human Perception and Performance, 31(6), 1404–1415. 16366798 10.1037/0096-1523.31.6.1404

[bibr34-17470218211037128] Reber R. Schwarz N. Winkielman P. (2004). Processing fluency and aesthetic pleasure: Is beauty in the perceiver’s processing experience? Personality and Social Psychology Review, 8(4), 364–382. 15582859 10.1207/s15327957pspr0804_3

[bibr35-17470218211037128] Roos L. E. Lebrecht S. Tanaka J. W. Tarr M. J. (2013). Can singular examples change implicit attitudes in the real-world? Frontiers in Psychology, 4, Article 594. 10.3389/fpsyg.2013.00594 PMC3763199 24046756

[bibr36-17470218211037128] Rosseel Y. (2012). lavaan: An R package for structural equation modeling. Journal of Statistical Software, 48(2), 1–36.

[bibr37-17470218211037128] Schonberg T. Bakkour A. Hover A. M. Mumford J. A. Nagar L. Perez J. Poldrack R. A. (2014). Changing value through cued approach: An automatic mechanism of behavior change. Nature Neuroscience, 17(4), 625–630. 24609465 10.1038/nn.3673 PMC4041518

[bibr38-17470218211037128] Silver A. M. Stahl A. E. Loiotile R. Smith-Flores A. S. Feigenson L. (2020). When not choosing leads to not liking: Choice-induced preference in infancy. Psychological Science, 31(11), 1422–1429. 33006289 10.1177/0956797620954491

[bibr39-17470218211037128] Streicher M. C. Estes Z. (2015). Touch and go: Merely grasping a product facilitates brand perception and choice. Applied Cognitive Psychology, 29(3), 350–359.

[bibr40-17470218211037128] Whitman J. C. Zhao J. Roberts K. H. Todd R. M. (2018). Political orientation and climate concern shape visual attention to climate change. Climatic Change, 147(3–4), 383–394.

[bibr41-17470218211037128] Wispinski N. J. Gallivan J. P. Chapman C. S. (2020). Models, movements, and minds: Bridging the gap between decision making and action. Annals of the New York Academy of Sciences, 1464(1), 30–51. 30312476 10.1111/nyas.13973

[bibr42-17470218211037128] Wright L. Steptoe A. Fancourt D . (2020, October 21). What predicts adherence to COVID-19 government guidelines? Longitudinal analyses of 51,000 UK adults. MedRxiv. https://www.medrxiv.org/content/10.1101/2020.10.19.20215376v1

[bibr43-17470218211037128] Zajonc R. B. (1968). Attitudinal effects of mere exposure. Journal of Personality and Social Psychology, 9(2), 1–27.5667435

