# Supplementary material for: In the hands of the beholder: Wearing a COVID-19 mask is associated with its attractiveness
Source: Q J Exp Psychol (Hove). 2021 Aug 11;75(4):598–615. doi: 10.1177/17470218211037128 (PMC8915245; doi:10.1177/17470218211037128)
Supplement: sj-docx-1-qjp-10.1177_17470218211037128 – Supplemental material for In the hands of the beholder: Wearing a COVID-19 mask is associated with its attractiveness [file sj-docx-1-qjp-10.1177_17470218211037128.docx]

Table SI1: Demographic and sociocultural survey questions used in the study, in the order presented to the participants. The questions used in the PCA are marked in the table.

| **Qn** | **Question** | **Used in the PCA** |
| --- | --- | --- |
| 1 | Gender | v |
| 2 | Age | v |
| 3 | Native language |  |
| 4 | Number of foreign languages known | v |
| 5 | Country of residence |  |
| 6 | State/province of residence |  |
| 7 | City/town of residence |  |
| 8 | Time lived in the current residence (1 = just visiting, 6 = more than 10 years) | v |
| 9 | How would you describe annual income of your household? (1=low, 2=middle, 3=upper-middle, 4=upper) | v |
| 10 | Are you a student? (1=yes, 2=no) | v |
| 11 | What is the longest time you have lived in one city? | v |
| 12 | Do you identify with a religion? (1=yes, 2=no) | v |
| 13 | What is your religion? |  |
| 14 | Frequency of participating in religious practices/service |  |
| 15 | Do you consider yourself an independent thinker? (1=yes, 5=no) | v |
| 16 | Do you tend to be an anxious person? (1=yes, 5=no) | v |
| 17 | Preferred news sources |  |
| 18 | Are you interested in politics? (1=no, 5=yes) | v |
| 19 | Do you enjoy debating political issues? (1=no, 3=yes) | v |
| 20 | Politically, would you describe yourself as left or right wing? (1=left, 9 = right) | **v** |
| 21 | Politically, would you describe your close friends and family as left or right wing? (1=left, 9 = right) | v |
| 22 | How often are you checking the news on COVID-19? (1 = once a week, 5 = more than 5 times a day) | v |
| 23 | How often are you checking news unrelated to COVID-19? (1 = once a week, 5 = more than 5 times a day) | v |
| 24 | How often were you checking the news before the COVID-19 pandemic? (1 = once a week, 5 = more than 5 times a day) | v |
| 25 | Have you had COVID-19 (coronavirus)? (1-3 yes or maybe, 4=no) | v |
| 26 | Do you consider yourself to be especially vulnerable to COVID-19? (1 = no, 4 = yes) | v |
| 27 | Do you live in close contact with a person who is especially vulnerable to COVID-19? (1 = no, 4 = yes) | v |
| 28 | Before 2020, have you been using hygienic gloves on a regular basis? (1= never, 4 = very often) |  |
| **Qn** | **Question** | **Used in the PCA** |
| 29 | These days, are you using hygienic gloves on a regular basis? (1= never, 4 = very often) |  |
| 30 | If you use hygienic gloves, when did you start using them? (1= never, 4 = very often) |  |
| 31 | Before 2020, did you use a protective face mask on a regular basis? (1= never, 4 = very often) |  |
| 32 | These days, are you using protective face masks on a regular basis? |  |
| 33 | When did you start using a protective face mask? |  |
| 34 | Before 2020, did you use hand sanitizer on a regular basis? |  |
| 35 | These days, are you using hand sanitizer on a regular basis? |  |
| 36 | When did you start using hand sanitizer? |  |
| 37 | Before 2020, did you use a diving mask? |  |
| 38 | These days, do you use a diving mask? |  |
| 39 | I put on a mask every time I leave the house. (1= never, 4 = very often) |  |
| 40 | I put on a mask only when I am in a crowded place. (1= never, 4 = very often) |  |
| 41 | I put on a mask when going into a store / supermarket / pharmacy. (1= never, 4 = very often) |  |
| 42 | When I'm outside, I have a mask with me. (1= never, 4 = very often) |  |
| 43 | I estimate the situation and only put the mask on when I think it is necessary. (1= never, 4 = very often) |  |
| 44 | I put on a mask even when I exercise. (1= never, 4 = very often) |  |
| 45 | Types of masks used |  |
| 46 | Number of masks owned since the pandemic onset |  |
| 47 | Number of masks owned now |  |
| 48 | How often do you use a face mask? (1=never, 6=every day) |  |
| 49 | Are you working from home? (1=never, 5=always) | v |
| 50 | Is a mask mandatory for your work? (1=yes, 2=no) | v |
| 51 | On how many days in the past week have you met other people (offline)? (0-7) | v |
| 52 | During a typical week, on how many days do you meet with people offline? (0-7) | v |
| 53 | Do you think the danger of COVID-19 has been (1=underestimated, 9=overestimated) | v |
| 54 | Do you think the usefulness of protective face masks against COVID-19 infection has been (1=underestimated, 9=overestimated) | v |
