# Supplementary material for: In the hands of the beholder: Wearing a COVID-19 mask is associated with its attractiveness
Source: Q J Exp Psychol (Hove). 2021 Aug 11;75(4):598–615. doi: 10.1177/17470218211037128 (PMC8915245; doi:10.1177/17470218211037128)
Supplement: sj-docx-2-qjp-10.1177_17470218211037128 – Supplemental material for In the hands of the beholder: Wearing a COVID-19 mask is associated with its attractiveness [file sj-docx-2-qjp-10.1177_17470218211037128.docx]

Table SI2: List of 26 questions included in the PCA, with (1) loadings from the exploratory principal component analysis computed on the data from the first wave of testing (July-August 2020) and (2) loadings from the confirmatory principal component analysis of questionnaire responses from October 2020 (replication). Question order is based on loadings in the exploratory PCA (ascending), question numbers refer to those listed in the Table SI1.

| **Question** | **Loadings from Exploratory PCA** | | | **Loading from Confirmatory PCA** |  |
| --- | --- | --- | --- | --- | --- |
|  | **1** | **2** | **3** |  |  |
| **Component 1: Belief in the danger of COVID** | | | | | |
| Conservative political orientation (SI Q20) | **0.72** | -0.373 | 0.045 | **0.71** |  |
| Doubt in the danger of COVID-19 (SI Q53) | **0.677** | -0.37 | -0.064 | **0.86** |  |
| Doubt in the the usefulness of face masks against COVID-19 (SI Q54) | **0.636** | -0.304 | -0.071 | **0.81** |  |
| Political orientation of friends & family (SI Q21) | **0.573** | -0.201 | -0.018 | **0.573** |  |
| Age (SI Q2) | **0.562** | 0.158 | 0.439 | **0.278** |  |
| Do you tend to be an anxious person? (reversed, SI Q16) | **0.524** | -0.097 | -0.036 | **0.312** |  |
| Do you identify with a religion? (reversed, SI Q12) | **-0.424** | 0.139 | 0.053 | **-0.265** |  |
| Gender | **-0.376** | -0.108 | 0.026 | **-.223** |  |
| Income of the household | **0.248** | -0.068 | -0.12 | **.142** |  |
| How long have you been living in the current residence? | **0.207** | -0.074 | 0.172 | **.018** |  |
| **Component 2: Interest in politics** | | | | | |
| Frequency of checking news unrelated to COVID-19 (SI Q23) | 0.274 | **0.775** | -0.002 | **0.909** |  |
| Interest in politics (SI Q18) | 0.133 | **0.756** | -0.044 | **0.517** |  |
| Frequency of news checking before COVID-19 (SI Q24) | 0.351 | **0.704** | 0.009 | **0.805** |  |
| Frequency of COVID news checking (SI Q22) | 0.246 | **0.698** | 0.038 | **0.662** |  |
| Do you enjoy debating political issues? (SI Q19) | 0.147 | **0.544** | -0.178 | **0.301** |  |
| Vulnerability of close people to COVID-19 | -0.138 | **0.29** | 0.182 | **.109** |  |
| Do you consider yourself an independent thinker? | -0.106 | **-0.268** | 0.112 | **-.223** |  |
| **Component 3: Exposure to COVID** | | | | | |
| Frequency of meeting people offline on a typical week (SI Q52) | 0.283 | 0.109 | **-0.726** | **-0.954** |  |
| Frequency of meeting people offline during past week (SI Q51) | 0.253 | 0.11 | **-0.689** | **-0.898** |  |
| Is a mask mandatory for your work? (reversed, SI Q50) | 0.005 | -0.029 | **0.561** | **0.336** |  |
| Are you a student? (reversed, SI Q10) | 0.376 | 0.029 | **0.417** | **0.123** |  |
| Vulnerability to COVID-19? (SI Q26) | -0.093 | 0.333 | **0.356** | **0.132** |  |
| Are you working from home? (SI Q49) | -0.029 | 0.043 | **0.272** | **0.255** |  |
| N of foreign languages known | -0.119 | 0.006 | **-0.3** | **-.194** |  |
| Have you had COIVD-19? | 0.096 | 0.04 | **0.101** | **-.007** |  |
