# Supplementary material for: In the hands of the beholder: Wearing a COVID-19 mask is associated with its attractiveness
Source: Q J Exp Psychol (Hove). 2021 Aug 11;75(4):598–615. doi: 10.1177/17470218211037128 (PMC8915245; doi:10.1177/17470218211037128)
Supplement: sj-docx-3-qjp-10.1177_17470218211037128 – Supplemental material for In the hands of the beholder: Wearing a COVID-19 mask is associated with its attractiveness [file sj-docx-3-qjp-10.1177_17470218211037128.docx]

Table SI3

Zero-order correlations between emotional ratings of masks, self-reported wearing practices, and individual differences indexed by the three PCA components, first wave of data collection (July-August 2020, N = 379)

|  | Mask attractiveness | Neutral mask attractiveness | Mask arousal | Neutral mask arousal | Mask wearing frequency | Months of mask wearing (in months) |
| --- | --- | --- | --- | --- | --- | --- |
| Mask attractiveness | 1 | .518^**^ | .388^**^ | .404^**^ | .260^**^ | .312^**^ |
|  |  | .000 | .000 | .000 | .000 | .000 |
| Neutral mask attractiveness | .518^**^ | 1 | .230^**^ | .623^**^ | -.038 | .051 |
|  | .000 |  | .000 | .000 | .460 | .318 |
| Mask arousal | .388^**^ | .230^**^ | 1 | .426^**^ | .163^**^ | .078 |
|  | .000 | .000 |  | .000 | .001 | .129 |
| Neutral mask arousal | .404^**^ | .623^**^ | .426^**^ | 1 | .021 | .035 |
|  | .000 | .000 | .000 |  | .691 | .500 |
| Mask wearing frequency | .260^**^ | -.038 | .163^**^ | .021 | 1 | .591^**^ |
|  | .000 | .460 | .001 | .691 |  | .000 |
| Months of mask wearing (months) | .312^**^ | .051 | .078 | .035 | .591^**^ | 1 |
|  | .000 | .318 | .129 | .500 | .000 |  |
| Distrust in the danger of COVID | -.142^**^ | .251^**^ | -.015 | .188^**^ | -.338^**^ | -.226^**^ |
|  | .006 | .000 | .773 | .000 | .000 | .000 |
| Interest in politics | .010 | -.103^*^ | .076 | .003 | .219^**^ | .231^**^ |
|  | .853 | .045 | .140 | .957 | .000 | .000 |
| Situational exposure to COVID | -.197^**^ | -.054 | -.008 | -.091 | -.044 | -.018 |
|  | .000 | .298 | .883 | .077 | .391 | .730 |
| **. Correlation is significant at the 0.01 level (2-tailed). | | | | | | |
| *. Correlation is significant at the 0.05 level (2-tailed). | | | | | | |

Table SI4

Zero-order correlations between emotional ratings of masks, self-reported wearing practices, and individual differences indexed by the three PCA components, second wave of data collection (July-August 2020, N = 330)

|  | Mask attractiveness | Neutral mask attractiveness | Mask arousal | Neutral mask arousal | Mask wearing frequency | Months of mask wearing (in months) |
| --- | --- | --- | --- | --- | --- | --- |
| Mask attractiveness | 1 | .391^**^ | .260^**^ | .382^**^ | .244^**^ | .262^**^ |
|  |  | .000 | .000 | .000 | .000 | .000 |
| Neutral mask attractiveness | .391^**^ | 1 | .224^**^ | .562^**^ | -.041 | .105 |
|  | .000 |  | .000 | .000 | .463 | .326 |
| Mask arousal | .260^**^ | .224^**^ | 1 | .406^**^ | .124^*^ | .162^**^ |
|  | .000 | .000 |  | .000 | .025 | .003 |
| Neutral mask arousal | .382^**^ | .562^**^ | .406^**^ | 1 | .085 | .134^*^ |
|  | .000 | .000 | .000 |  | .126 | .015 |
| Mask wearing frequency | .244^**^ | -.041 | .124^*^ | .085 | 1 | .620^**^ |
|  | .000 | .463 | .025 | .1026 |  | .000 |
| Months of mask wearing (months) | .262^**^ | .105 | .162^**^ | .134^*^ | .620^**^ | 1 |
|  | .000 | .059 | .003 | .015 | .000 |  |
| Distrust in the danger of COVID | -.218^**^ | .210^**^ | -.006 | .115^*^ | -.333^**^ | -.378^**^ |
|  | .000 | .000 | .921 | .038 | .000 | .000 |
| Interest in politics | -.051 | .077 | .140^*^ | .017 | -.002 | .054 |
|  | .354 | .160 | .011 | .761 | .967 | .329 |
| Situational exposure to COVID | -.016 | -.073 | .002 | -.060 | .039 | .095 |
|  | .770 | .187 | .969 | .276 | .487 | .086 |
| **. Correlation is significant at the 0.01 level (2-tailed). | | | | | | |
| *. Correlation is significant at the 0.05 level (2-tailed). | | | | | | |
