# Supplementary figures and images for: In the hands of the beholder: Wearing a COVID-19 mask is associated with its attractiveness
Source: Q J Exp Psychol (Hove). 2021 Aug 11;75(4):598–615. doi: 10.1177/17470218211037128 (PMC8915245; doi:10.1177/17470218211037128)

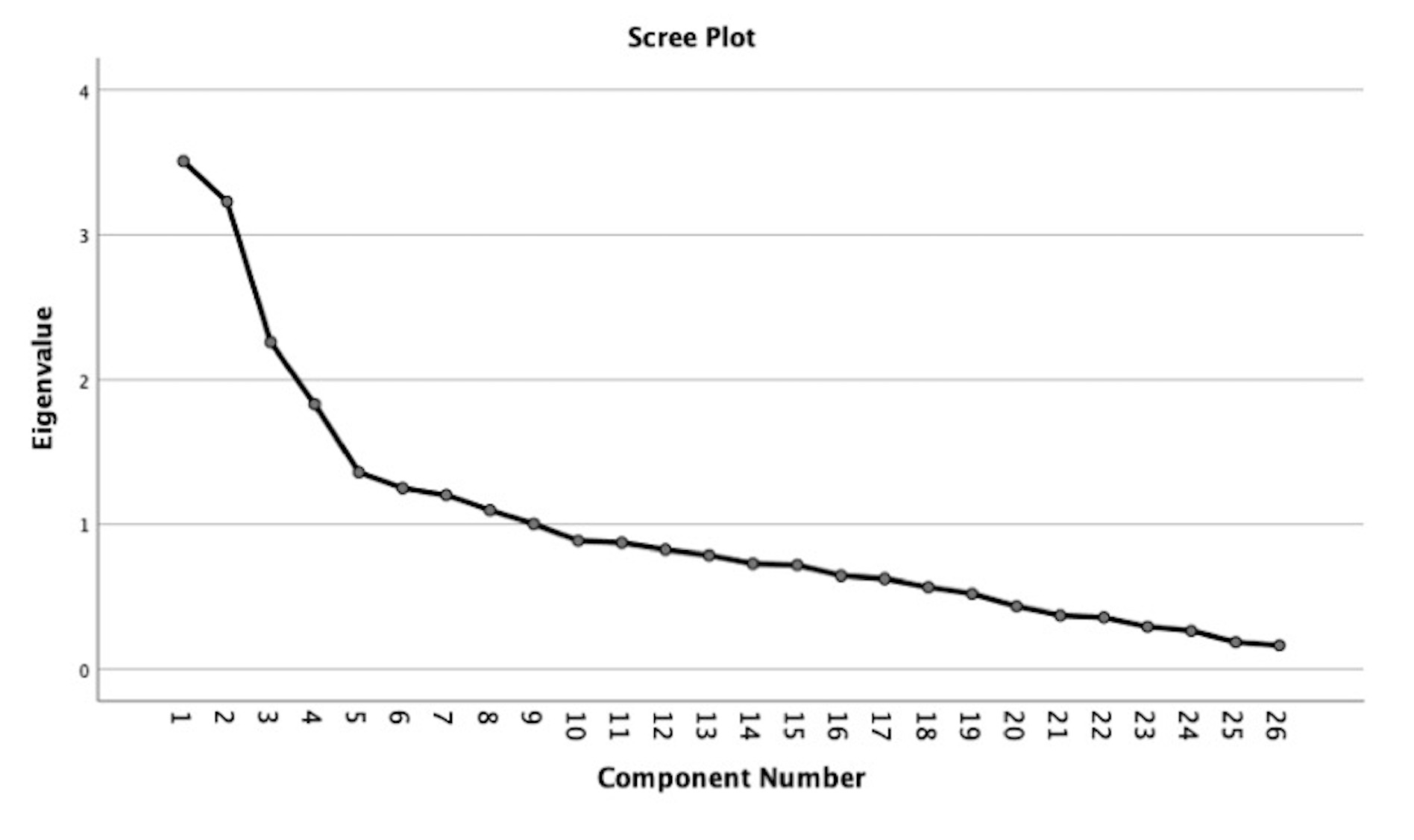

Supplement: sj-jpeg-4-qjp-10.1177_17470218211037128 – Supplemental material for In the hands of the beholder: Wearing a COVID-19 mask is associated with its attractiveness [file sj-jpeg-4-qjp-10.1177_17470218211037128.jpeg]
